# Amplitude Integrated Electroencephalography and Continuous Electroencephalography Monitoring Is Crucial in High-Risk Infants and Their Findings Correlate With Neurodevelopmental Outcomes

**DOI:** 10.3389/fped.2021.691764

**Published:** 2021-08-03

**Authors:** Inn-Chi Lee, Syuan-Yu Hong, Yi-Ho Weng, Yi-Ting Chen

**Affiliations:** ^1^Division of Pediatric Neurology, Department of Pediatrics, Chung Shan Medical University Hospital, Taichung, Taiwan; ^2^Institute of Medicine, School of Medicine, Chung Shan Medical University, Taichung, Taiwan; ^3^Division of Pediatrics Neurology, Department of Pediatrics, Children's Hospital, China Medical University, Taichung, Taiwan

**Keywords:** EEG monitoring, aEEG, neonatal encephalopathy, hypoglycemic, seizures

## Abstract

**Background:** To evaluate seizure diagnosis in sick infants in the neonatal intensive care unit (NICU) based on electroencephalography (EEG) monitoring combined with amplitude integrated electroencephalography (aEEG).

**Methods:** We retrospectively reviewed EEG and aEEG findings and determined their correlations with neurodevelopmental outcomes at the age of >1 year in 65 patients with diagnosed seizures, encephalopathy, or both.

**Results:** Seizure identification rate was 43.1%. The rate in nonstructural groups (hypocalcemic, hypoglycemic, and genetic seizures) was 71.4%, which was higher (*p* < 0.05) than the rate of 35.3% of structural brain lesion group [hypoxic–ischemic encephalopathy (HIE) and congenital brain structural malformation]. The aEEG background correlating with neurodevelopmental outcomes had 70.0% positive prediction value (PPV), 65.5%% negative prediction value (NPV), 67.7% specificity, and 67.9% sensitivity (*p* < 0.005). The aEEG background strongly (PPV, 93.8%; *p* < 0.005) correlated with the outcomes in HIE. For genetic seizures, the detected rate was high. The ictal recordings for the nonstructural seizures revealed downflected on the aEEG background initially, which differed from the structural lesion.

**Conclusions:** EEG monitoring combined with aEEG can detect seizures, facilitating early treatment. EEG changes during seizures could exhibit delta-theta waves with or without clinical seizures in patients with brain lesions. In non-structural etiologies (hypocalcemic and *KCNQ2* seizures), aEEG initially exhibited lower background during seizures that could aid in differentiating these EEG changes from those of other etiologies. The aEEG background was correlated with neurodevelopmental outcome and exhibited high PPV but not NPV in neonatal HIE.

## Introduction

Infants who require admission to the neonatal intensive care unit (NICU) are at a particularly high risk of brain disorders, encephalopathy, and seizure. The diagnosis of seizures in sick infants based only on clinical presentations is often inaccurate and not timely (1). Non-convulsive seizures and non-convulsive status epilepticus are common in infants with encephalopathy, and the sick infants have a high non-convulsive seizure risk (1). Early diagnosis and rapid treatment are critical for the long-term prognosis of neonatal seizures (2). Therefore, identifying an effective method for seizure diagnosis and the early initiation of appropriate therapy is imperative because it enables the prompt initiation of appropriate therapy.

Neonatal seizures and encephalopathy are typically detected on the basis of the patient's clinical history; neurological examination, particularly in unconsciousness newborns; and sudden clinical events suspected of seizures. Further examination includes the electroencephalography (EEG), computed tomography (CT) and magnetic resonance imaging (MRI) findings, and specific clinical features, all of which can offer a clue for diagnosis, albeit insufficient. In some cases of hereditary or genetic-related seizures, genetic studies can be used, especially in critically ill patients where an imaging study is challenging. However, performing further neurological examination and initiating early treatment is still warranted.

Amplitude integrated electroencephalography (aEEG) and continuous conventional video-EEG monitoring can define seizures well, delineate the non-convulsive seizure pattern, and detail the EEG findings during seizure onset, which might be effective in rapidly diagnosing neonatal seizures or early encephalopathy (3–5). The aEEG is widely used in the NICU as a standard of care for neonatal encephalopathy (6, 7). Prolonged video-EEG monitoring is useful for diagnosing and differentiating neonatal seizure or encephalopathy with diverse etiologies and facilitates the accurate detection of electroclinical syndromes (2, 3, 8–12). Ictal EEG outcome associated with various etiologies are rarely reported. The detection of the same pattern on aEEG and EEG monitoring suggests that this is a distinct aEEG seizure pattern in specific etiology like *KCNQ2* (13, 14). that can facilitate early treatment, including oxcarbazepine and phenytoin.

We hypothesized that aEEG and EEG patterns in seizures differ from those of seizures caused by various etiologies in NICU. Therefore, we investigated the aEEG and EEG monitoring in the sick infants with various etiologies to observe the EEG changes and correlate them with neurodevelopmental outcomes.

## Patients and Methods

### Study Design

We retrospectively reviewed cases recorded during 2017–2019 of children with various etiologies who were admitted to an NICU at the age of 0 days to 4 months.

### Inclusion and Exclusion Criteria

Of 1,029 patients admitted to the NICU, we enrolled 105 (10.2%) patients with reported seizures, encephalopathy, or both. We arranged aEEG and EEG monitoring for all patients. The etiologies included hypoxic–ischemic encephalopathy (HIE), congenital brain structural malformations, premature infants with acquired brain lesions, hypocalcemic or hypoglycemic seizures, seizure-like events, and genetic seizures. Further confirmation of etiologies is willing to correlate the EEG monitoring findings. After we excluded premature infants with acquired brain lesions and seizure-like events, 65 patients were enrolled on the basis of their clinical presentation (perinatal history, MRI, or metabolic examination). Patient charts were retrospectively reviewed. Notably, aEEG and conventional EEG monitoring were performed in all the 65 cases.

### EEG Study

All patients underwent conventional video-EEG monitoring (NicoletOne, Nicolet vEEG, Natus, Seattle, WA, USA) at least one time and at least 24-h. The EEG findings were determined using referential and bipolar recording, which included longitudinal and transverse montages. Continuous EEG monitoring was applied with a modified 10–20 international placement montage with eight scalp electrodes at F3, F4, C3, C4, P3, P4, O1, and O2. Monitoring was conducted for at least 24 h. Measurements from an EEG monitor were processed in NicoletOne software (Nicolet vEEG, Natus, Seattle, WA, USA) to obtain aEEG data (C3–P3 and C4–P4).

The aEEG backgrounds were classified as normal amplitude, with the upper margin of aEEG band of aEEG activity >10 mV and the lower margin >5 mV, sleep-wake cycling (SWC) matched to the age, and no electrographic seizures; moderately abnormal amplitude, with discontinuous activity and the upper margin of aEEG band activity >10 mV and the lower margin ≤ 5 mV or normal amplitude with electrographic seizures; and severe abnormal amplitude, with the upper margin of the band of aEEG activity <10 mV and the lower margin ≤ 5 mV without SWC, including burst-suppression (discontinuous activity with lower margin at 0–1 μV constantly and a burst amplitude >25 μV), flat trace, and continuous low voltage (continuous very low amplitude activity at about 5 μV or below 5 μV) (11, 15, 16). Seizure was defined at least one of [i.] a period of sudden increase in voltage accompanied by a narrowing of the band of aEEG activity, associated with video-confirmed clinical visual seizures (2, 3, 15) and [ii.] EEG seizure without clinical observation of seizure. EEG seizure without clinical observation were defined as paroxysmal activity on EEG that were not caused by artifacts or external environment interference. When the aEEG measurements indicated a seizure diagnosis, the findings were further confirmed by a corresponding EEG feature. Paroxysmal activity on EEG was defined as paroxysmal activity with a duration of at least 10 s. The aEEG and EEG findings were reviewed for prompt diagnosis of the etiology of neonatal seizure or encephalopathy. For patients with confirmed seizures, a series of examinations, including head ultrasound and MRI, were performed.

### Neurodevelopmental Outcomes Study

The Bayley Scales of Infant and Toddler Development, Third Edition (Bayley-III) was adopted to evaluate neurodevelopmental outcomes at the age of >1 year. The Bayley-III scores were interpreted as follows: normal, ≥85; mild, ≥70 and <85; moderate, ≥55 and <70; and severe, <55 (17, 18). The cognitive and motor subscales in Bayley-III scores were used to express the neurodevelopmental outcomes.

### Statistical Analysis

The statistical differences between different groups were analyzed using an independent *t*-test on SPSS (version 14.0; SPSS Institute, Chicago, IL, USA). Significant differences were evaluated using an independent *t*-test or a chi-square statistic test. Significance was set at *p* < 0.05. If sample distribution was non-parametric, a Mann-Whitney *U*-test was performed.

## Results

### Demographic Data

Table 1 presents the demographic data of the groups with different etiologies. The mean age of EEG monitors and aEEG examinations was 14.0 ± 25.8 days (median, 3 days) in total 65 cases. In the HIE group (*n* = 41), the mean age of EEG monitors and aEEG examinations was 2.6 ± 0.5 days (median, 3 days) after birth. The gender and age at examination did not differ significantly among different groups. However, the number of antiepileptic medications prescribed for groups with genetic seizures were significantly higher (*p* < 0.005) than it was for other groups, which indicated that seizures because of those etiologies were more refractory, particularly genetic seizures, and required more antiepileptic drugs for seizure control.

**Table 1:** Demographic data of 65 infants.

	**Hypoxic*-*ischemic encephalopathy (*N* = 41)**	**Congenital brain structural malformations (*N* = 10)**	**Hypocalcemic or hypoglycemic seizures (*N* = 7)**	**Genetic seizures (*N* = 7)**	**Total (*N* = 65)**
Post-natal age at the time of study (day) (mean ± SD)	2.6 ± 0.5	65.7 ± 33.4	7.1 ± 2.3	14.0 ± 4.5	14.0 ± 25.8
Gender					
Male	21 (51.2%)	6 (60.0%)	4 (57.1%)	5 (71.4%)	36 (55.4%)
Female	20 (48.8%)	4 (40.0%)	3 (42.9%)	2 (28.6%)	29 (44.6%)
Number of patients with detected seizures^±^	15 (36.6%)	3 (30.0%)	4 (57.1%)	6 (85.7%)	28 (43.1%)
Number of anti-seizure drugs at electroencephalography (EEG) study^#^					
0	21(51.2%)	8 (80.0%)	0 (0.0%)	0 (0%)	29 (44.6%)
1	17 (41.5%)	0 (0.0%)	5 (71.4%)	0 (0%)	22 (33.8%)
2	2 (4.9%)	0 (0.0%)	0 (0.0%)	5 (71.4%)	7 (10.8%)
>2	1 (2.4%)	2 (20.0%)	2 (28.6%)	2 (28.6%)	7 (10.8%)

± *The ratio of patients with detected seizures in non-structural lesion of brain was 71.4% (hypoglycemia, hypoglycemic seizure, and genetic seizures) were higher (p <0.05; odds ratio 5.14; 95% confidence interval: 1.47 to 17.92) than the ratio in the structural brain lesion (HIE, congenital brain structural malformation), which were 35.3%*.

# *The number of antiepileptic medications prescribed for genetic-group seizures were significantly higher (p <0.05) than for the other groups*.

*SD indicates standard deviation*.

### Ratio of Patients With Detected Seizures

The seizure identification among the 65 cases was 28 (43.1%). The detection rate of seizures among the 65 cases was 43.1%. The ratio was highest in 6 (85.7%) of the 7 patients with genetic seizures (6 are *KCNQ2* seizures, 1 is *GABRB3*); 15 (36.6%) of the 41 cases of HIE; 3 (30%) of the 10 cases with congenital structural brain malformations; 4 (57.1%) of the 7 hypocalcemic or hypoglycemic seizures (Table 1). The seizure identification rate in patients with non-structural brain lesions was 71.4% (hypoglycemia, hypoglycemic seizure, and genetic seizures), which was higher (*p* < 0.05; odds ratio 5.14; 95% confidence interval: 1.47 to 17.92) than the rate of 35.3% in patients with structural brain lesions (HIE and congenital brain structural malformation).

**Figure 1:**
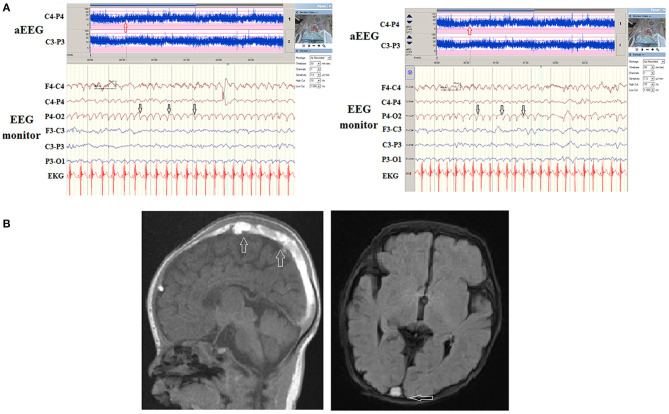
**(A)** A patient with stage I hypoxic*-*ischemic encephalopathy (HIE). However, during the automated electroencephalography (aEEG) and electroencephalography (EEG) monitor, recurrent paroxysmal focal delta waves were noticed right posterior regions (white arrows) on EEG monitor and a notch on aEEG (red arrows). **(B)** The magnetic resonance imaging (MRI) and magnetic resonance venography **(**MRV) exhibited diffuse thrombosis (arrows) of superficial great vein with small hemorrhage of right occipital lobe.

Overall, 41 patients had HIE, including 19 with Sarnat staging of ≥2, received therapeutic hypothermia. Of these 41 patients with HIE, 15 (36.6%) were detected to have clinical or EEG seizures on EEG monitoring. Among 22 patients with stage I HIE, one was detected with EEG seizures without clinical seizures, and subsequently diagnosed diffusely sinus thrombosis in the superficial great sinus vein (Figure 1). The seizure pattern on aEEG was that of a sudden rise in the lower and upper margins, with an associated EEG pattern of delta-theta waves in focal brain region. The aEEG background strongly correlated with the HIE outcome, with a positive prediction rate (PPV) of 93.8%, negative prediction rate (NPV) of 63.2%, specificity of 92.3%, and sensitivity of 68.2%.

**Figure 2:**
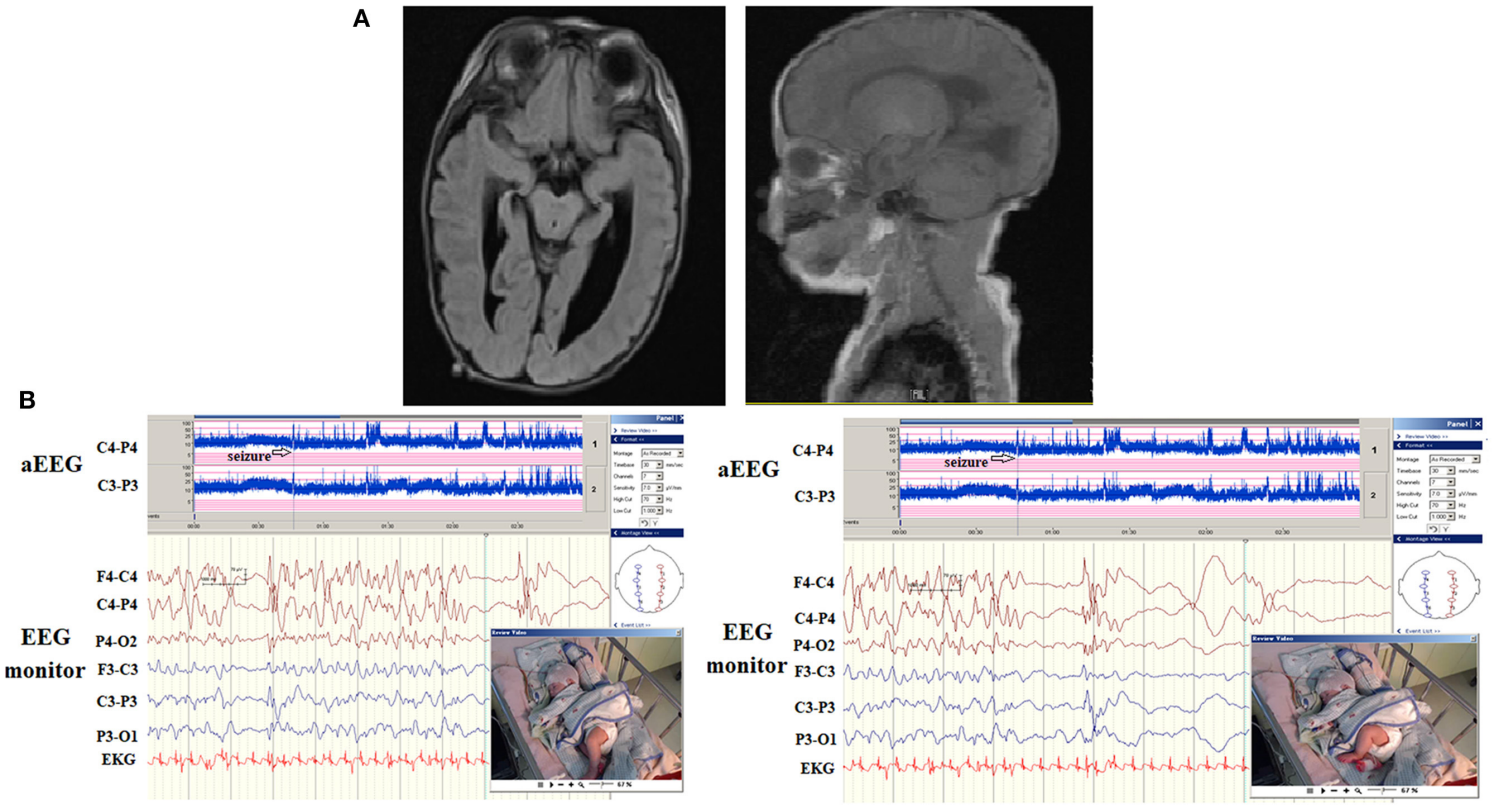
**(A)** A patient had a congenital structural brain malformation with asymmetrical and irregular size of ventricles, and suspected focal cortical dysplasia. His first seizure onset was at 2 months of age with bilateral alternative clonic seizures of legs. **(B)** The aEEG detected frequent seizures and electroencephalography (EEG) monitoring detected irregular spikes occurring in the right and left hemispheres (upper, arrows) associated with left and right clonic seizures independently.

Three (30%) of the 10 cases with congenital structural brain anomalies were detected to have seizures on EEG monitoring. Ten cases with congenital brain anomalies included two tuberous sclerosis (TS), two corpus callosum dysplasia (one agenesis, one corpus callosum dysplasia), one Pelizaeus-Merzbacher disease, one lissencephaly, one neurofibromatosis type 1, one congenital bilateral ventriculomegaly with focal cortical dysplasia and two holoproencephaly. One patient with congenital bilateral ventriculomegaly with focal cortical dysplasia was detected to have focal seizures at 2 months with associated aEEG and EEG abnormalities along with clinical focal seizures (Figure 2).

**Figure 3:**
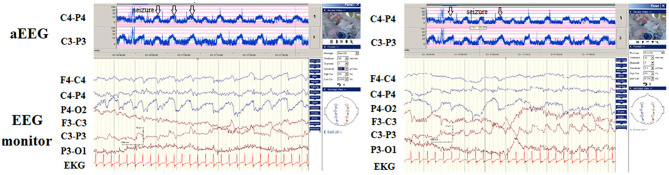
A patient presented with hypoglycemic seizures at neonatal day 2. His seizures did not remit after treatment with several antiepileptic drugs. The automated electroencephalography (aEEG) demonstrated frequent seizures associated with ictal paroxysmal activity on electroencephalography (EEG) monitoring (arrows). She had severe developmental delay at the age of 1 year. The magnetic resonance imaging (MRI) results demonstrated diffuse brain atrophy.

**Figure 4:**
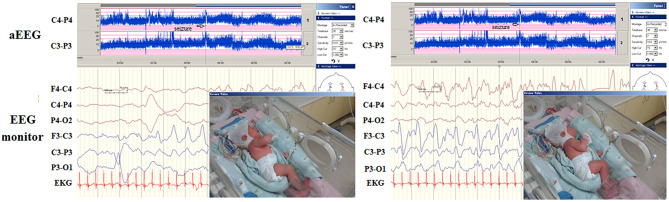
Hypocalcemic seizure since day 8 of birth in one newborn. The automated electroencephalography (aEEG) and electroencephalography (EEG) monitoring revealed three seizures within 1 h (arrows) with the aEEG background initially lower, then upflected. His calcium level was 6.8 mg/dL (ion calcium, 0.71 mg/dL).

Among the seven patients (including five with hypocalcemic and two with hypoglycemic seizures), four patients (57.1%) were detected to have seizures on aEEG and EEG monitoring. The seizures in two patients with hypoglycemic seizures were frequent and were finally associated with secondary brain injuries, as noted on MRI (brain atrophic changes; Figure 3). In patients with hypocalcemic seizures, the aEEG demonstrated an initially lower background, which then upflected; however, the background between seizures was normal (Figure 4). The aEEG background in all cases correlated with neurodevelopmental outcomes at the age of 1 year.

Seven patients had genetic seizures confirmed using genetic study, including six *KCNQ2* and one *GABRB3*, without structural brain abnormalities. Six *KCNQ2* seizures included five neonatal epileptic encephalopathies (EE) and one benign familial neonatal convulsion (BFNC). The seizure identification rate among *KCNQ2* EE was 100%. One patient with *GABRB3* infantile EE presented with first seizures at age 2 months. *GABRA*3 causes frequent prominent epileptic spasms and atypical absence seizures. In *KCNQ2* seizures, the ictal seizures displayed on aEEG had a stereotypical triphasic pattern, which first downflected on the aEEG background, then upflected, and downflected subsequently. Notably, the ictal EEG could be synchronous in both hemispheres or localized in one hemisphere.

### The aEEG Findings Correlated With Cognitive and Motor Neurodevelopmental Outcomes

The patients' aEEG background activity was classified as normal, moderate, or severe, and its correlation with neurodevelopmental outcomes identified through the Bayley-III at the age of >1 year was evaluated (Table 2). The mean duration of neurodevelopmental follow-up was 32.9 ± 11.3 months, ranged from 18 to 53 months. For the 59 cases with Bayley-III results, aEEG background activity was correlated with cognitive neurodevelopmental outcomes, with a PPV of 70.0%, NPV of 65.5%, specificity of 67.7%, and sensitivity of 67.9% (*p* = 0.005; odds ratio = 4.43; 95% confidence interval, 1.51 to 12.99; Table 2); aEEG background activity was correlated with motor neurodevelopmental outcomes, with a PPV of 82.8%, NPV of 66.7%, specificity of 70.6%, and sensitivity of 80.0% (*p* = 0.000; odds ratio = 9.60; 95% confidence interval, 2.88 to 32.05 in motor subscales; Table 2). The aEEG background significantly correlated with the outcomes, particularly in patients with HIE (*p* = 0.001) (Table 2). In the HIE group, PPV was 93.8%, indicating that the moderate and severe aEEG background could predict worse outcomes.

**Table 2:** The Amplitude integrated electroencephalography (aEEG) and Electroencephalography (EEG) monitoring, and neurodevelopmental outcomes with Bayley-III evaluated at the age of at least older than 1 year in different groups.

	**Hypoxic*-*ischemic encephalopathy (*N* = 35)^@^**	**Congenital brain structural malformations^※^ (*N* = 10)**	**Hypocalcemic or hypoglycemic seizures^$^ (*N* = 7)**	**Genetic seizures^±^ (*N* = 7)**	**Total (*N* = 59)**
Background of aEEG					
Normal	16 (45.7%)	6 (60.0%)	5 (71.4%)	1 (14.3%)	28 (47.4%)
Moderate	14 (40.0%)	3 (30.0%)	1 (14.3%)	6 (85.7%)	24 (40.7%)
Severe	5 (14.3%)	1 (10.0%)	1 (14.3%)	0 (0.0%)	7 (11.9%)
Cognitive outcomes with Bayley-III after 1 year old					
Normal	18 (51.4%)	0 (0.0%)	5 (71.4%)	1 (14.3%)	24 (40.7%
Mild	4 (11.4%)	7 (70.0%)	0 (0.0%)	0 (0.0%)	11 (18.7%)
Moderate	7 (20.0%)	1 (10.0%)	1 (14.3%)	1 (14.3%)	10 (16.9%)
Died or severe	6 (17.2%)	2 (20.0%)	1 (14.3%)	5 (71.4%)	14 (23.7%)
Positive prediction value**^#^**	93.8%^%^	66.7%	100.0%	100%	70.0%^&^
Negative prediction value**^#^**	63.2%^%^	25.0%	100.0%	100%	65.5%^&^
Specificity**^#^**	92.3%^%^	57.1%	100.0%	100%	67.7%^&^
Sensitivity**^#^**	68.2%^%^	33.3%	100.0%	100%	67.9%^&^
Motor outcomes with Bayley-III after 1 year old					
Normal	18 (51.5%)	6 (60%)	5 (71.4%)	1 (14.3%)	30 (50.8%)
Mild	5 (14.3%)	1 (10%)	0 (0.0%)	1 (14.3%)	7 (11.9%)
Moderate	6 (17.1%)	1 (10%)	1 (14.3%)	0 (0.0%)	8 (13.6%)
Died or severe	6 (17.1%)	2 (20%)	1 (14.3%)	5 (71.4%)	14 (23.7%)
Positive prediction value**^#^**	88.2%^%^	50.0%	100.0%	100.0%	82.8%^&^
Negative prediction value**^#^**	66.7%^%^	25.0%	100.0%	83.3%	66.7%^&^
Specificity**^#^**	71.4%^%^	50.0%	100.0%	50.0%	70.6%^&^
Sensitivity**^#^**	85.7%^%^	25.0%	100.0%	100.0%	80.0%^&^

@ *Among 41 patients with hypoxic-ischemic encephalopathy, 6 patients were without Bayley-III, including 3 stage I, 2 stage II, and 1 stage III*.

※ *Ten patients with congenital brain structural malformations included 2 tuberous sclerosis, 2 corpus callosum dysplasia, 1 lissencephaly, 1 Pelizaeus-Merzbacher disease, 1 neurofibromatosis type I, 2 holoprosencephaly, and 1 congenital bilateral ventriculomegaly with focal cortical dysplasia*.

$ *Seven patients included 2 hypoglycemic seizures and 5 hypocalcemic seizures*.

± *Seven patients with genetic seizures included 6 KCNQ2 seizures (5 KCNQ2 epileptic encephalopathy and one with benign familial neonatal convulsion) and 1 GABRB3 epileptic encephalopathy*.

# *The background of aEEG to correlate with worse or equal to moderate outcome at corrected age of 1 year old*.

& *Total cases, p = 0.005; odds ratio = 4.43; 95% confidence interval, 1.51 to 12.99 in cognitive subscales;. p = 0.000; odds ratio = 9.60; 95% confidence interval, 2.88 to 32.05 in motor subscales*.

% *HIE group, p = 0.001; odds ratio = 25.71; 95% confidence interval, 2.97 to 222.92 in cognitive subscales; p = 0.001; odds ratio = 15.00; 95% confidence interval, 2.69 to 83.70 in motor subscales*.

## Discussion

**Figure 5:**
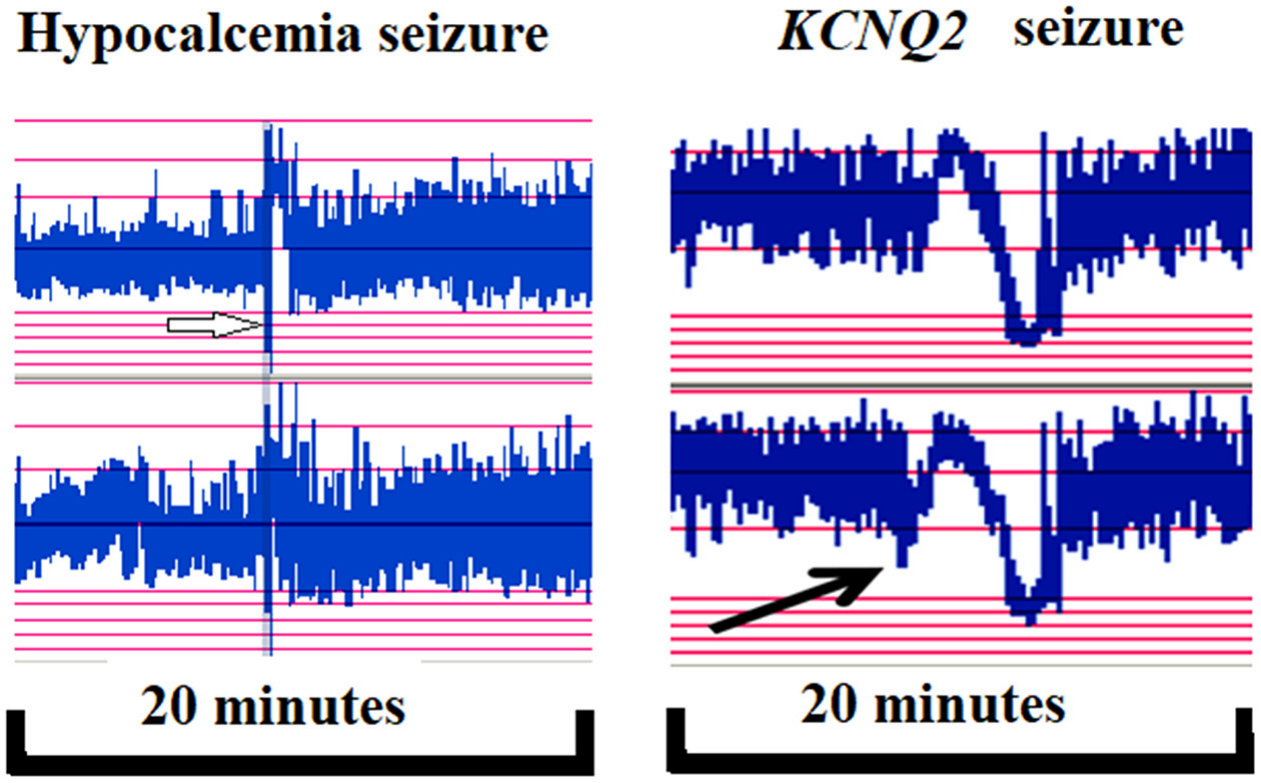
In hypocalcemic (left, white arrow) and *KCNQ2* (right, black arrow) seizures, the ictal seizures on automated electroencephalography (aEEG) displayed a stereotypical triphasic pattern in aEEG, which was first downflected of the aEEG background, then upflected and then downflected.

A significant contribution of this study is its delineation of aEEG and EEG monitoring features and its evaluation of these features' correlations with neurodevelopmental outcomes at the age of >1 year in patients with seizures or encephalopathy of various etiologies. We demonstrated the aEEG and EEG monitoring to be valuable tools in detecting seizures and correlating with neurodevelopmental outcome. The morphology of ictal aEEG and EEG monitoring in patients with hypocalcemic and genetic seizures without structural brain anomalies differed from that of ictal aEEG and EEG of seizures related to structural brain anomalies that are often associated with secondary brain injury or developmental brain anomalies. In neonatal seizures, the ictal EEGs generally demonstrate delta–theta waves during the seizure attack in structural brain anomalies. By contrast, hypocalcemic and genetic seizures initially exhibit a stereotypical triphasic pattern of aEEG background activity in which it is first downflected, then upflected, and then downflected again (Figure 5). Using the long-term EEG monitoring and aEEG can detect seizures in critically ill infants with high risk. In addition, EEG monitoring and aEEG can enable prompt diagnosis of the etiology of neonatal seizures and facilitate early initiation of effective treatment.

In patients with HIE, the PPV was as high as if the aEEG were performed after day 1; this finding is compatible with that of a study that indicated that the aEEG background can predict the outcome when the day of examination is after day 1 (19). For genetic seizures, the seizure identification rate was high In genetic seizures, similar to that of *KCNQ2* EE, the detection of seizure was high owing to the frequent and refractory seizures in these patients. The aEEG backgrounds in *KCNQ2* EE patients normal amplitude with electrographic seizures, and were correlated with long-term neurodevelopmental outcomes; however, the “developmental epileptic encephalopathy” that highlights genetic dysfunction is an independent factor in determining the outcomes despite cases with *KCNQ2* seizures being seizure-free beyond newborn age.

The outcomes of neonatal encephalopathy with EEG monitoring and aEEG were the most studied (5, 19–21). Prognostic value of aEEG in neonates with high risk of neurological sequelae in 250 infants highlighted that severely abnormal aEEG predicted adverse neurological outcome with a high sensitivity of 70.2%, specificity of 87.1%, PPV of 75.6%, and NPV of 83.7% (20). However, the aEEG in these cases were performed before 6 h life, and not performed a day after day 1 like in our study. Furthermore, in 80 infants receiving therapeutic hypothermia, the pattern of background evolution was more reliable than the background pattern at discrete intervals to distinguish outcome (19). Background aEEG activity depression at the age of 3 and 4 days and delay in the appearance of cyclic activity were associated with worse MRI scores predictive of worse long-term outcome (21), similar to that observed in our study. In a prospective study of 100 children with acute encephalopathy, Abend et al. noted electrographic seizures in 46%, electrographic status in 19%, with exclusively non-convulsive seizures in 32% (5). Only 50, 87, and 97% of these were detected within the first hour, the first 24 h, and the first 48 h, respectively.

Because most seizures are EEG alone (subclinical, non-convulsive) and clinical manifestations might be subtle, several clinicians consider EEG data, including conventional EEG or aEEG, as valuable tools in identifying seizures in those patients in NICU (5). Nevertheless, the aEEG yields highly variable sensitivities and specificities, and therefore, cannot be recommended as the mainstay for neonatal seizure diagnosis and management (22). Hence, a combination of EEG monitoring and aEEG is an alternative, particularly in critically ill patients in NICU. The interictal aEEG findings revealed relatively better background in most seizures related to non-structural etiologies revealed by an MRI, such as hypocalcemia and *KCNQ2* EE, compared with the seizures linked to those with other etiologies. Seizures caused by hypocalcemia or *KCNQ2* mutations occur most frequently in the first 2 weeks after birth. Notably, interictal EEG findings, which are typically single or multiple focal spikes, have low specificity for determining etiologies. The specific pattern of burst suppression in EEG findings is typically attributable to a severe etiology, such as severe HIE or *KCNQ2* or metabolic EE. Typically, the focal spikes and paroxysmal activity are non-specific.

Our study noted seven neonatal seizures owing to genetic mutations, including six *KCNQ2* seizures and one *GABRB*3 seizure, without any lesions detected on MRI. Nevertheless, owing to the advancements in genetic etiology examination, the neonatal seizures could be confirmed using a genetic study like whole exon sequencing. Notably, the characteristics of different genetic seizures are noteworthy. Neonatal genetic seizures that are heterogeneous in genetic etiology and often associated with ultra-rare genetic causes (23). The characteristics of EEG findings can be helpful in defining the cause of seizure—either non-genetic or genetic—even associate it with different genes, including *KCNQ2, SCN8A, SCN2A*, and *SCN1A*. In *KCNQ2* EE, the interictal and ictal phases are considered more severe than the EEG in BFNC. The EEG features of seizures because of *KCNQ2* mutations are different based on the location of the affected amino acid of *KCNQ2* mutations, accounting for its phenotype (BFNC or EE). *KCNQ2* mutations in the pore loop between S5 and S6 contains a highly conserved selectivity filter that controls potassium ion permeability, yielding the characteristic EEG findings (13, 14). The *KCNQ2* pathogenic mutations cause Kv7.2 current inhibition causing neuron hyperexcitability and excessive neuronal discharges. When these cells fire signals together, the ictal EEG records fast activity. However, in patients with secondary brain injury causing neuron cell necrosis, the EEG reveals no initial fast activity but reveal delta-theta waves during the seizure attack. The *SCN8A* epilepsy was reported to be generalized symmetric tonic seizures with autonomic signs, varying from subtle to marked clinical manifestations in 80% of seizures (24). Nearly 50% of *SCN8A* seizures started in the neonatal period with normal interictal EEG and slowly emerging seizures and myoclonic jerks (25). The others would have rapid general seizure onset with interictal burst-suppression EEG (25). *STXBP1* seizure onset is more heterogeneous and often sudden. Seizure onset was at 6 months of age on average, ranging from 1 day to 6 months. The interictal EEG of *STXBP1* EE displayed a suppression-burst pattern in 50% of the cases. Therefore, differentiation of the EEG patterns of genetic and non-genetic seizures is crucial for clinicians in facilitating early diagnosis and initiating empirical treatment.

Nonetheless, this study had some limitations. Because neonatal seizures are rare, we presented a limited number of cases of different etiologies to analyze the aEEG and EEG monitoring findings; hence, our findings might be biased. The data had some bias related to different etiologies, particularly for genetic, hypocalcemic, and hypoglycemic seizures, which comprise fewer cases. We had recordings of stereotypical ictal EEG of neonatal seizures. However, real-time recording of the ictal seizures from different etiologies was challenging. Nevertheless, the effects are crucial. Therefore, studies with more number of cases are warranted. The number of electrodes used for the EEG monitoring of newborns was reduced to compensate for their small head circumference. We used a modified system with eight scalp electrodes, one each at F3, F4, C3, C4, P3, P4, O1, and O2. A reduced montage has been reported to have higher sensitivity and specificity for the detection of neonatal seizures (26, 27). Furthermore, in the HIE group, the time of aEEG and EEG monitoring is critical, which in our study was a day after day 1, which could provide reliable outcome prediction during the era of rescued hypothermia therapy. Some neonatal patients have EEG seizures without exhibiting clinical seizures; this phenomenon is probably correlated with poorer outcomes compared with those without any clinical seizures or EEG seizures. Moreover, seizure frequency is not necessarily correlated with the outcome; for example, benign KCNQ2-associated seizures have favorable outcomes. We endeavor to further study the correlation between seizure frequency and neurodevelopmental outcome.

## Conclusion

The conventional EEG monitoring can help detect the seizures early and facilitate timely treatment initiation. Based on the ictal EEG findings, continuous delta-theta waves with or without clinical seizures are often observed in brain lesional etiology. The non-structural etiologies, such as hypocalcemia and neonatal *KCNQ2* seizures, had a specific feature that could help differentiate the EEG of these seizures from the other etiologies. The aEEG background was correlated with neurodevelopmental outcome and exhibited a high PPV but not NPV in neonatal HIE. Nevertheless, conventional EEG monitoring combined with aEEG could enable prompt diagnosis of seizures and facilitate early initiation of effective treatment.

## Data Availability Statement

The raw data supporting the conclusions of this article will be made available by the authors, without undue reservation.

## Ethics Statement

The studies involving human participants were reviewed and approved by The Internal Review Board of Chung Shan Medical University Hospital provided the ethics approval (IRB #: CS2-14003), and the study was performed per the relevant guidelines. Written informed consent for participation was not required for this study in accordance with the national legislation and the institutional requirements. Written informed consent for participation was not provided by the participants' legal guardians/next of kin because: Written informed consent for participation was not required for this study in accordance with the national legislation and the institutional requirements. Written informed consent was not obtained from the minor(s)' legal guardian/next of kin for the publication of any potentially identifiable images or data included in this article.

## Author Contributions

I-CL conceptualized the research idea, devised the methodology, and provided guidance and supervision during trace analysis. Y-HW and Y-TC carried out amplitude-integrated encephalography trace analysis as well as clinical data collection. I-CL and S-YH drafted the manuscript and reviewed the manuscript. All authors contributed to the article and approved the submitted version.

## Conflict of Interest

The authors declare that the research was conducted in the absence of any commercial or financial relationships that could be construed as a potential conflict of interest.

## Publisher's Note

All claims expressed in this article are solely those of the authors and do not necessarily represent those of their affiliated organizations, or those of the publisher, the editors and the reviewers. Any product that may be evaluated in this article, or claim that may be made by its manufacturer, is not guaranteed or endorsed by the publisher.
